# Effects of habit formation interventions on physical activity habit strength: meta-analysis and meta-regression

**DOI:** 10.1186/s12966-023-01493-3

**Published:** 2023-09-12

**Authors:** Haoming Ma, Aoqi Wang, Runyuan Pei, Meihua Piao

**Affiliations:** School of Nursing, Chinese Academy of Medical Sciences & Peking Union Medical College, No 33 Ba Da Chu Road, Shijingshan District, Beijing, 100144 People’s Republic of China

**Keywords:** Habit formation, Meta-analysis, Physical activity, Automaticity

## Abstract

**Background:**

Interventions aimed at promoting physical activity (PA) behavior through habit formation pathways are gaining popularity, as they differ from conventional interventions that rely on intention pathways. Past research has established a positive correlation between PA habits and behavior. However, the efficacy of current interventions designed to form PA habits and improve PA automaticity is not yet fully ascertained. Additionally, the intervention components that significantly impact the effectiveness of these interventions are yet to be determined.

**Methods:**

This systematic review was conducted in accordance with the Preferred Reporting Items for Systematic Reviews and Meta-Analyses guidelines. We conducted a search of three databases (PubMed, Embase, and Cochrane Library) from January 2000 to December 2022, with a focus on interventions for developing PA habits. Two independent authors conducted paper selection, quality assessment, data extraction, and coding of behavior change techniques (BCTs). The effect size of interventions was calculated using standardized mean difference. Subgroup analyses were carried out based on follow-up duration, delivery method, sample characteristics, and theory. Furthermore, we employed meta-regression to investigate the association between BCTs and PA habits.

**Results:**

Ten eligible studies with relatively high quality were included in the final data set. Characteristics of studies varied in intervention sample and delivery way. The habit formation interventions significantly increased PA habit (SMD = 0.31, 95% CI 0.14—0.48, *P* < .001) compared to the control groups. Subgroup analysis demonstrated that the duration of follow-up ≤ 12 weeks have a higher effect size on PA habit than the duration > 12 weeks. Meta-regression revealed that problem solving has a significant positive association with effectiveness improvement (β = 0.36, 95% CI 0.17–0.55), while social reward is linked with a reduction in effectiveness (β = -0.40, 95% CI -0.74–0.06).

**Conclusions:**

Our findings reveal that habit formation interventions are effective in fostering PA habit. Future studies could leverage the insights form this study to optimize the intervention design and achieve better effectiveness.

**Supplementary Information:**

The online version contains supplementary material available at 10.1186/s12966-023-01493-3.

## Background

Regular physical activity (PA) has many health benefits, including improved fitness, well-being, sleep quality, and decreased risk of chronic diseases across all ages [1, 2]. However, less than 20% of individuals follow the recommended guidelines for PA, posing a major public health hazard [2, 3]. Therefore, effective interventions are imperative to promote PA behavior change.

Currently, health behavior change approaches predominantly rely on Reasoned Action Theory [4], including the Theory of Planned Behavior [5] and the Health Action Process Approach [6]. These models prioritize the intention construct as the primary driver of behavior and modify it and its antecedents, such as attitude and self-efficacy, to promote PA behavior [7]. However, despite the significant association between intention and PA, a considerable discrepancy known as the "intention-behavior gap" exists between them, suggesting other significant factors may influence health behavior [7–9].

Dual-process theories propose two pathways to explain health behavior: the reflective pathway, which involves cognitive effort and deliberation, as illustrated by reasoned action accounts, and the intuitive pathway, which is triggered by context and occurs automatically without consciousness [10]. Habit has emerged as a key determinant of automatic behavior, formed through repeated practice in specific contexts and habit formation interventions were based on this pathway to foster health behavior through this cue-response associations [11].

Traditionally, habit has been measured by past behavioral frequency, but this measure cannot distinguish between reasoned and automatic action since, in stable contexts, both repeated deliberation and habit can produce the same pattern of frequent behavior [12]. To better capture the underlying processes that drive habitual behavior, the Self-Report Habit Index (SRHI) and Self-report behavioral automaticity index (SRBAI) were developed [13, 14]. The SRHI assesses automaticity, frequency, and relevance to self-identity, while the SRBAI focuses on the fundamental nature of habit-behavior relationships in a concise, four-item automaticity subscale. PA automaticity refers to the degree to which a specific PA behavior is performed in a manner that is effortless and unconscious, without requiring conscious awareness or effort. Currently, the SRHI and SRBAI are the most frequently employed scales for measuring PA habit [4].

Recent meta-analyses used the SRHI and SRBAI scales to examine the correlation between habit strength and health behaviors, highlighting the significant impact of habit on health behavior [4, 15]. Previous literature reviews have described habit formation interventions for promoting health behavior, including targeted behaviors and behavior change techniques (BCTs) [16, 17]. Although these studies demonstrate the significant impact of PA habit strength on behavior, the effectiveness of existing habit formation interventions in enhancing PA habit strength, as well as the intervention components that substantially impact efficacy, remain uncertain.

Few studies have quantitatively synthesized the impact of PA habit formation interventions on PA habit, and the characteristics of these interventions and their impact on efficacy have not been fully understood. Therefore, the purpose of this research is to address these gaps by (a) systematically compiling the characteristic of habit-forming interventions, (b) pooling their effect size, (c) identifying the active components (BCTs) utilized, and (d) determining the individual impact of these components on the efficacy of the interventions.

## Methods

This systematic review followed the PRISMA (Preferred Reporting Items for Systematic Reviews and Meta-Analyses) guidelines [18], and the study protocol was registered in the PROSPERO database with registration ID CRD42022373159 (see Additional file 1).

## Information sources and search strategies

**Fig. 1:**
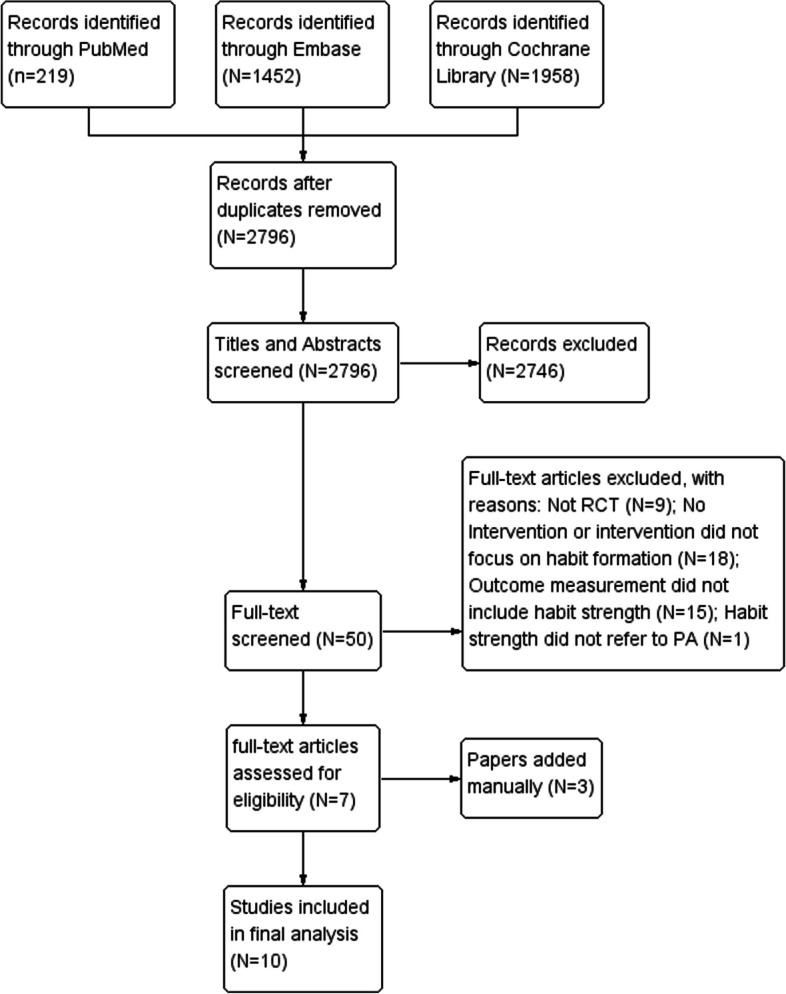
PRISMA flow chart of the search strategy

In the current article, three electronic health databases (PubMed, Embase, and Cochrane Library) were searched in the period from January 2000 to December 2022 focusing on the interventions for forming PA habits. Moreover, we retrieved 4 systematic reviews to prevent undiscovered related literature [1, 4, 15, 16]. The search procedures of this study were shown in Fig. 1.

The search terms we used were presented in Additional file 2, which principally amalgamated synonyms related to the intervention (habit formation) with synonyms related to the outcomes (PA), and these terms were mapped to medical subject headings (MeSH) when accessible. Literature was deemed eligible for further screening only if they were published in the English language, in a full-text format in peer-reviewed journals, and reported habit strength scores of PA before and after habit formation interventions.

We used EndNote X9.2 to remove repetitive articles. Moreover, we checked the reference lists of relevant reviews and papers to avoid any missing studies.

### Eligibility criteria

#### Intervention

The studies included should aim to develop automatic associations between specific cues and PA behavior through interventions. Any approaches designed to facilitate this cue-triggered association in PA are considered habit formation interventions. This study focuses on forming new PA habits, such as promoting stair climbing in an office setting, rather than breaking unwanted habits like sedentary behavior at home. Hence, studies that do not support new habit formation or only focus on reducing sedentary habits were excluded.

#### Studies design

Only studies in randomized controlled trial design were included as eligible.

#### Population

Healthy people or people with specific health conditions were all accepted in this study. We excluded studies with participants who have limited mobility or severe cognitive impairment.

#### Control or comparator

The studies included featured control groups or waiting-control groups that did not receive habit formation interventions, or received non-habit formation interventions such as education on the benefits of PA. For instance, only the data from the first four months of Piao et al.'s work were analyzed, as the habit-forming intervention was also given to the control group from the fifth to the twelfth week, rendering data after four months invalid [19].

#### Outcome measure

Included studies should measure habit strength using SRHI or SRBAI for a specific PA behavior at baseline and follow-up. Studies that solely used behavior frequency as an outcome measurement were excluded since it cannot capture the automaticity of a habit [11].

The integration of the SRHI and the SRBAI is rational due to their shared purpose of measuring habitual behavior through self-reported scales, with a focus on the automaticity and stability of individual behavior. Moreover, the measurement indicators of SRHI and SRBAI demonstrate high consistency and comparability, making their integration viable for enhancing the reliability and validity of analysis [13]. The combined results can more clearly reflect the strength and automaticity of habitual behavior, and provide more reliable quantitative data suitable for meta-analysis.

Habit strength of other related behaviors is not eligible, as seen in the exclusion of a study that focused on forming the habit of wearing activity trackers, which is not equivalent to PA behavior [20].

#### Study selection

All references were screened for eligibility by two independent reviewers (HM and AW) according to the eligibility criteria through two rounds of screening. The first round involved screening search results based on the title, abstract, and keywords, while the second round involved full-text screening of studies suspected to be eligible or where eligibility was unclear. Inconsistencies during each round of screening were resolved in a lab meeting led by a third reviewer (MP) until a consensus was reached.

#### Data extraction

Two reviewers (HM and AW) independently performed the initial data extraction using a standardized framework developed specifically for this review. If any discrepancies in data extraction were identified, a lab meeting was held by a third reviewer (MP) to recheck the original study. The data extracted included the basic study characteristics such as the title, authors, publication year, and country of origin; study participants including sample size, age, and sex ratio; study design including intervention duration, content of intervention group and control group, the BCTs used to form new habits, duration of follow-up, and theoretical foundation; outcome measures including the measurement used to evaluate habit formation and its timing; and key results including the mean and SD for SRHI or SRBAI of PA at baseline and follow-up.

#### Bias assessments

The risk of methodological bias was independently assessed by 2 authors according to the “Cochrane Collaboration’s tool for assessing the risk of bias” [21]. Disagreements between reviewers were discussed among the study team until a consensus was finally reached. To ascertain publication bias, a funnel plot and Egger's test were performed.

#### Synthesis of results

The meta-analysis entailed the pooling of habit strength indicators, including means and standard deviations (SDs), utilizing the Review Manager software (Version 5.4.1, The Nordic Cochrane Centre). Notably, the approach adopted in this analysis involved the integration of data from both baseline and final measurement points to calculate the distinct impacts of interventions on the intervention group relative to the control group, particularly in instances where studies featured multiple measurement points.

In light of the missing data, such as the specific SDs of the SRHI, we initially employed statistical conversion methods, such as the conversion of confidence intervals (CI) to SDs. Subsequently, we attempted to obtain the necessary information by reaching out to the corresponding author. If these attempts proved futile, we ultimately excluded the affected papers from the final synthesis.

In accordance with Cochrane's recommendations, we employed the I^2^ statistic to determine the presence of heterogeneity in our analysis [22]. To interpret the effect sizes, we referred to Cohen's work, which stipulates that effect sizes of 0.20, 0.50, and 0.80 denote small, medium, and large effects, respectively [23].

Subgroup analyses were conducted based on the following criteria: 1) intervention duration (≤ 12 weeks vs. > 12 weeks); 2) theory-based interventions (theory-based vs. non-theory-based); 3) intervention channel (offline-based vs. online-based); 4) samples with obvious condition (with condition vs. without condition).

The associations between different BCTs and habit formation were examined using random effects meta-regression. To enhance accuracy of the results, further control for the confounder was perform and the confounders was chosen based on the results of subgroup analyses (chosen when specific characteristic of intervention or sample have a significantly impact on intervention effectiveness).

Meta-regression analysis, subgroup analyses, and funnel plot charting were performed using IBM SPSS Statistics 28.0 under the guidance of Sen et al. [24]. Review Manager 5.4.1 was utilized for the extraction of information, assessment of risk of bias, generation of figures, and synthesis of effect sizes.

#### Effect size calculation

To calculate the overall impact of the habit formation intervention on PA habit strength, we utilized Cohen's d to standardize and align the SRHI and SRBAI scales. The standardized effect sizes were then combined to obtain the mean and confidence interval, allowing us to assess the collective impact of multiple studies [25].

Given the considerable heterogeneity arising from differences in study populations and intervention designs, we utilized a random effects model to consolidate the effect sizes across the included studies [26].

### Behavior change techniques coding

Two reviewers (HM, AW) independently encoded intervention BCTs utilizing Michie et al.'s taxonomy of 93 BCTs v1 [27], after completing online training on how to encode the taxonomy (https://www.bct-taxonomy.com/) to ensure accurate and dependable coding of the active ingredients for the included interventions. Any disagreement between the two reviewers was discussed within the study team until unanimity was eventually achieved.

## Results

### Study selection

Figure 1 illustrates that our initial database search yielded 2796 articles. Through the elimination of duplicate articles and a two-round screening process (abstract and full-text), we identified 10 articles that met the eligibility criteria for inclusion in the meta-analysis. In addition, we identified three relevant articles through manual searches.

### Study characteristics

Table 1 presents the salient features of the 10 studies that were included in this systematic review. All studies were randomized controlled trials, published in English from 2013 to 2022, and involved a total of 2,349 subjects. The sample sizes ranged from 45 [28] to 884 [29], with a broad spectrum of participant characteristics. Four studies targeted subjects with existing or potential health risks [28–31], while six studies enrolled subjects without evident health issues [19, 32–36]. Among the studies that targeted subjects with health risks, two focused on cardiovascular rehabilitation disease [28, 29], and one aimed to reduce the risk of cardiovascular disease [31]. One study aimed to target overweight people or patients with obesity [30]. Among the studies that targeted subjects without health issues, four studies recruited inactive individuals who failed to meet the recommended PA guidelines [32–34, 36]. The participants' age varied across the studies, ranging from children [33] to the elderly [36], with some studies targeting adults [32, 34] and university students and employees [35]. Additionally, one study was conducted in an office setting [19].

**Table 1:** Studies included in the review data set

Authors (Date) and country	Sample characteristics	Delivery way and delivery setting	Habit measurement, Total number of measurements and its time point	BCTs coding
Piao et al (2020) [19] South Korea	*N* = 106; office workers	Mobile online chatbot Volunteer	SRHI; 4 measurement point: 0–4 week	Habit formation, action planning, prompts/cues, Self-monitoring of behavior, Self-reward, Material reward (behaviour), Social reward, Goal setting (behavior)
Weyland et al (2022) [35] Germany	*N* = 132; participants of weekly exercise courses at a German university	Offline training Volunteer	SRHI; 10 measurement point: form week1-week10	Social support (emotional), Social reward, Prompts/cues, Self-reward
Carels et al. (2014) [30] USA	*N* = 59; overweight and obese adults (body mass index ≥ 27 kg/m2)	Offline training Volunteer	SRHI; 3 measurement point: at baseline, 12 week and 9-month	Habit formation, habit reversal, restructuring the physical environment, action planning, problem solving, nonspecific reward, avoidance/reducing exposure to cues for the behavior, prompt/cues, comparative imagining of future outcomes, social support (unspecified), self-monitoring of behavior
Storm et al. (2016) [31] Germany, Netherlands	*N* = 790; people who want to reduce their cardiovascular risk;	Online web-based intervention Volunteer	SRHI; 3 measurement point: baseline; 8 week and 12 week	Goal setting (behavior), problem solving, action planning, review behavioral goals, feedback on behavior, social support (unspecified), instruction on how to perform behavior, information on health consequences, social comparison, habit formation
Kaushal et al. (2018) [32] Canada	*N* = 97; inactive new gym members;	Offline workshop and a booster telephone follow-up Volunteer	SRBAI 2 measurement point: at baseline and 8 weeks	Habit formation, information about antecedents, action planning, prompts/cues, self-monitoring of behavior, problem solving, self-reward, behavioral practice/rehearsal
Western et al. (2022) [34] UK	*N* = 51; low to moderately active adults (PA level < 2.0)	Online web-based platform Volunteer	SRHI; 3 measurement point: at baseline, 6- and 12-week	Biofeedback, Feedback on outcome(s) of behaviour, Self-monitoring of behaviour, action planning, Discrepancy between current behaviour and goal, Goal setting (behaviour), Goal setting (outcome)
White et al. (2017) [36] UK	*N* = 91; older adults age 60–74 years, retired, and ≥ 6 h/day leisure sitting	An information booklet Primary health care	SRHI; 3 measurement point: at baseline, 8-week and 12-week	Goal setting (behavior), action planning, self-monitoring of behavior, self monitoring of outcomes of behavior, instructions on how to perform a behavior, information about health consequences, demonstration of the behavior, behavioral substitution, habit formation, habit reversal, graded tasks, restructuring the physical environment, adding objects to the environment, framing/reframing
Rhodes et al. (2021) [33] Canada	*N* = 102; parents whose children (aged 6–12 years) who did not meet international PA guidelines of at least 60 min of MVPA per day	Offline training Volunteer	SRBAI 4 measurement point: baseline, 6-week, 13-week, and 26-week	Instruction on how to perform the behaviour, Information about health consequences, Problem solving, action planning, Feedback on behaviour
Fournier et al. (2018) [28] France	*N* = 49; cardiovascular patients	Offline training and phone call interview Volunteer	SRBAI 5 measurement point: baseline, 5-, 7-, 9-, 12-month	Instructions on how to perform a behavior, Demonstration of the behavior, behavioral practice/rehearsal, habit formation, action planning, prompts/cues, social support (practical)
Fleig et al (2013) [29] Germany	*N* = 1166; individuals in cardiac and orthopedic rehabilitation	Telephone-delivery Primary health care	SRHI; 2 measurement point: at baseline and 18-month	Goal setting (outcome), action planning, focus on past success, self-monitoring of behavior, instructions on how to perform a behavior, demonstration of the behavior, behavioral practice/rehearsal, social support (unspecified)

*BCT* Behavior change techniques, *SRHI* Self-report habit index, *SRBAI* Self-report behavioral automaticity index

Furthermore, the interventions were delivered using various methods, including offline face-to-face training [28, 30, 32, 33, 35], web-based intervention [31, 34], mobile phone counseling [29, 32], paper booklet [36], and mobile phone chatbot [19]. Offline delivery was the predominant form, accounting for half of the interventions, while newer delivery methods are emerging, such as mobile phone chatbot [19]. Some studies used a combination of delivery methods [28, 32], and intervention durations ranged from four to twenty weeks.

### Risk of bias assessments

**Fig. 2:**
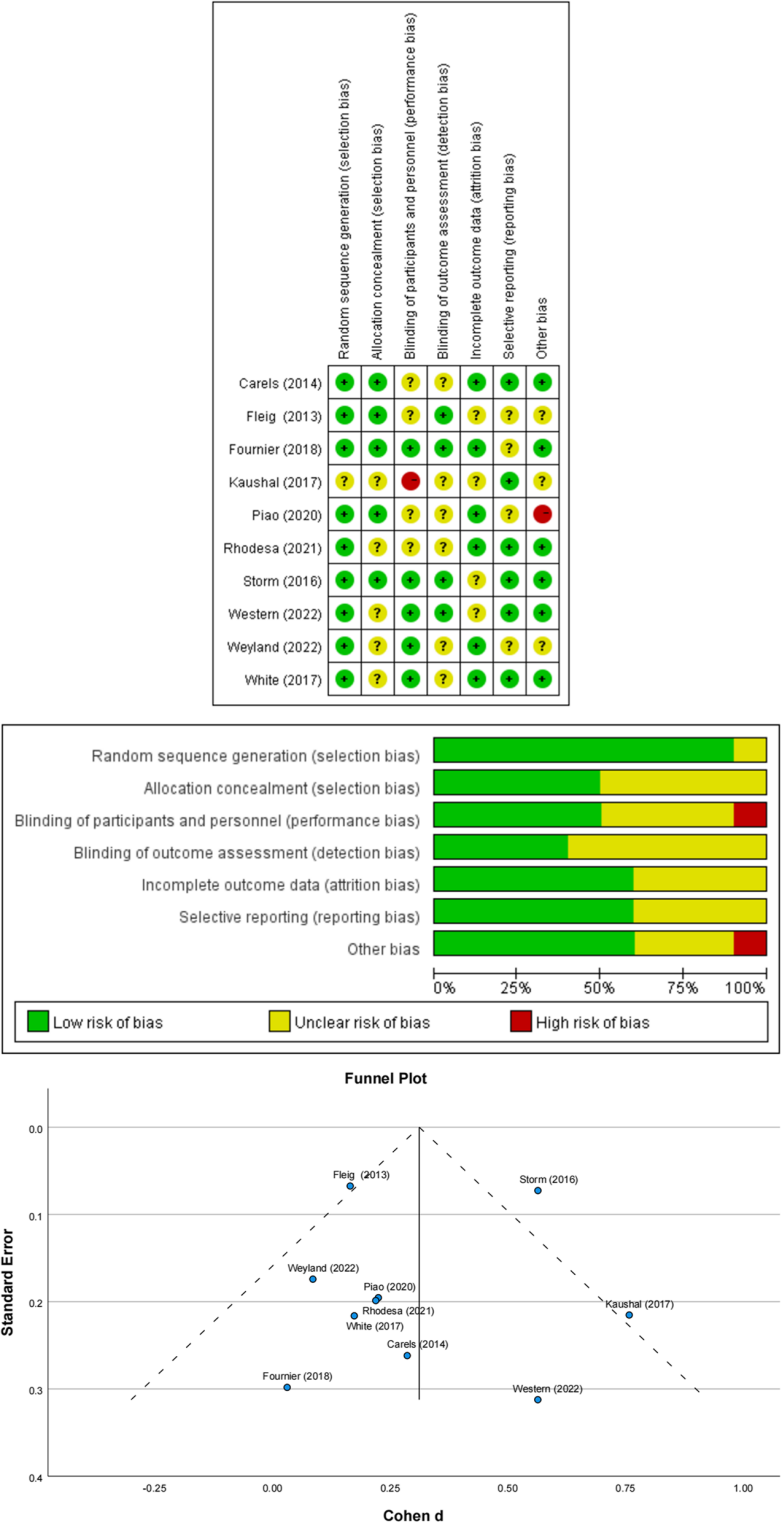
Risk of bias in individual studies

The risk of bias assessment results for the 10 studies included in this review are presented in Fig. 2, which shows that the quality of the eligible articles was relatively high. All accepted articles showed no evidence of potential selection bias, detection bias, attrition bias, or reporting bias. The funnel plot also appeared relatively symmetric, and the result of Egger's test (with an Egger's intercept of 0.34 and *p*-value of 0.12) indicated no significant risk of publication bias among these studies.

### Meta-analysis of habit formation intervention.

**Fig. 3:**
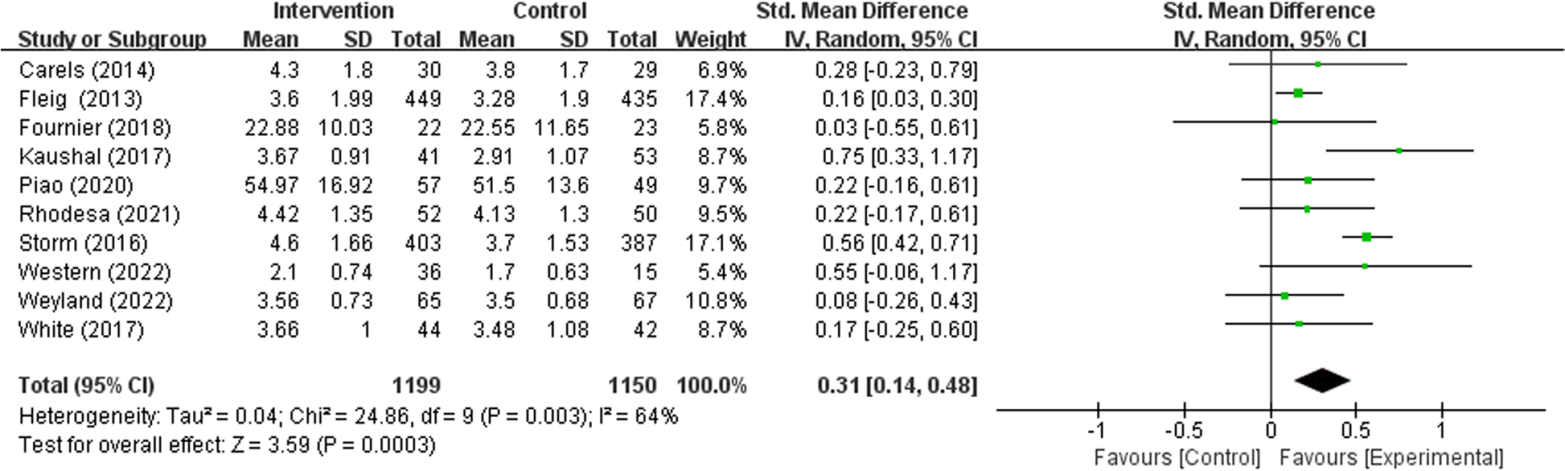
Forest plot of habit formation intervention studies effect on PA habit strength

The key result is depicted in the form of a forest plot in Fig. 3. A total of 10 studies, comprising a total of 2349 participants, revealed a significant difference in habit strength between the experimental group and the control setting (SMD = 0.31, Z = 3.59, 95% CI 0.14 to 0.48, *P* < 0.001; see Fig. 3). This effect should be considered a small to medium effect size according to the rule of thumb [37]. Concerning the heterogeneity tests, significant heterogeneity was revealed in the included study. (χ^2^ = 24.86, *P* = 0.003, I^2^ = 64%).

In the subgroup analyses, the delivery way (χ2 = 0.33, *P* = 0.56), whether sample with an obvious health issue (χ2 = 0.01, *P* = 0.95), and whether based on theory (χ2 = 0.60, *P* = 0.44) were all not significantly related to the intervention effectiveness. Among these, the pooled effect of the online delivery intervention is higher than compared with the offline delivery way (SMD 0.36 vs. 0.26); the pooled effect of interventions focused on the healthy individual is slightly lower than compared with those focused on the individual with an obvious health issue (SMD 0.30 vs. 0.32); the pooled effect of intervention based on theory is higher than compared with non-theory-based intervention (SMD 0.34 vs. 0.22).

Remarkably, the duration of follow-up significantly related to the effectiveness of interventions (χ^2^ = 6.38, *P* = 0.01). Through further analysis, the duration of follow-up ≤ 12 weeks (SMD 0.40, Z = 3.55, 95% CI 0.18 to 0.61, *P* < 0.001) have a higher effect size than the duration of follow-up > 12 weeks (SMD 0.17, Z = 2.81, 95% CI 0.05 to 0.29, *P* = 0.005).

### Meta-regression of BCTs

Meta-regression was conducted to explore the effect sizes of different BCTs used in the interventions included in our study. The BCTs with a frequency of at least 2 were considered for these analyses, and the results of the BCTs meta-regression are presented in Table 2. An additional file shows the full regression model of all BCTs [see Additional file 3]. The frequently used BCTs (identified in ≥ 50% of interventions) for promoting PA automaticity included action planning (9/10), habit formation (6/10), self-monitoring of behavior (6/10), and prompts/cues (5/10).

**Table 2:** Association between individual behaviour change techniques and effectiveness of habit formation interventions

BCTs	N	Associations (β, 90% CI) with effectiveness of habit formation interventions	
		Crude model	Model 1
Problem solving	4	0.36 (0.17–0.55) **	0.28 (0.04–0.52) *
Action planning	9	0.25 (-0.34–0.84)	0.42 (-0.03–0.87)
Habit formation	6	0.23 (-0.06–0.53)	0.15 (-0.23–0.51)
Feedback on behavior	2	0.22 (-0.12–0.56)	0.19 (-0.10–0.49)
Information about antecedents	2	0.19 (-0.31–0.69)	0.09 (-0.43–0.61)
Goal setting (behavior)	4	0.18 (-0.16–0.52)	0.04 (-0.46–0.53)
Goal setting (outcome)	2	0.08 (-0.52–0.37)	0.04 (-0.44–0.51)
Social support (unspecified)	3	0.07 (-0.32–0.46)	0.16 (-0.16–0.49)
Instruction on how to perform behavior	4	0.04 (-0.35–0.43)	0.04 (-0.32–0.40)
Self-reward	3	0.03 (-0.40–0.46)	-0.16 (-0.67–0.25)
Self-monitoring of behavior	6	0.02 (-0.40–0.46)	0.03 (-0.35–0.40)
Behavioral practice / Rehearsal	3	0.01 (-0.43–0.40)	0.11 (-0.33–0.55)
Prompts/cues	5	-0.05 (-0.44–0.34)	-0.16 (-0.52–0.2)
Restructuring the physical environment	2	-0.10 (-0.63–0.42)	-0.10 (-0.60–0.40)
Habit reversal	2	-0.10 (-0.63–0.42)	-0.10 (-0.60–0.40)
Information about health consequences	2	-0.14 (-0.63–0.35)	0.05 (-0.63–0.73)
Social reward	2	-0.20 (-0.66–0.26)	-0.40 (-0.74–0.06) *
Demonstration of the behavior	3	-0.25 (-0.60–0.09)	-0.17 (-0.60–0.25)

Crude model: not controlling for confounder

Model 1: controlling for confounder (Duration of follow-up)

*BCT* Behavior change techniques

^*^p ≤ 0.05

^**^p < 0.01

In the meta-regression, problem solving has a consistently significant positive association with improved habit strength (β = 0.36, 95% CI 0.17–0.55), even after controlling the significant confounder (β = 0.28, 95% CI 0.04–0.52). It is worth noting that our univariable meta-regression analysis did not reveal any significant correlation between social reward and reduced efficacy of interventions (β = -0.20, 95% CI -0.66–0.26). Nevertheless, after adjusting our model for confounding variables, a significant negative correlation was observed (β = -0.40, 95% CI -0.74–0.06).

## Discussion

### Overview of findings

Our systematic review of 10 studies on habit formation interventions aimed at promoting PA yielded valuable insights. This study, to the best of our knowledge, is the first meta-analysis to examine the efficacy of habit formation interventions for PA habits. The results of our analysis indicate that these interventions have a statistically significant impact on the PA habits strength, with a pooled effect size (calculated using a random effect model) of 0.31 (95% CI: 0.14–0.48) on PA automaticity. These findings are consistent with prior research, which has also suggested that habit formation interventions can effectively promote healthy behavior habits [16, 38].

Though all the included intervention studies adopted a habit formation approach, there still had a considerable heterogeneity, with an I^2^ value of 64%. This variation in results could potentially be attributed to disparities in intervention delivery methods, the BCTs employed, the duration of the intervention and follow-up periods, and the characteristics of the study population. This indicates that, habit formation intervention is still at its developing phase and do not have relatively well-acknowledged intervention guidelines or standers [39].

In the post hoc subgroup analysis, we found that the follow-up period has a significant impact on the effect measurement of the intervention. It was found that the effect size is smaller for interventions with a follow-up period of greater than 12 weeks compared to those with a follow-up period of 12 weeks or less. This consistent with the previous research on the law of automaticity change in newly formed habits, as habit strength reaches a plateau of automaticity after approximately 12 weeks and subsequently begins to gradually decline, ultimately stabilizing [40, 41]. Future research should consider this phenomenon when designing the follow-up duration.

Furthermore, our findings suggest that the delivery method used for habit formation interventions may have a significant impact on their effectiveness. Online delivery may be particularly advantageous, as it allows for more frequent and timely reminders and feedback, which can reinforce behavior change and promote habit formation [42]. The timely reminders can increase the likelihood of the planed behavior-cue enactment and promote habit formation through repeated behavior in a consistent environment [43]. Furthermore, in contrast to traditional offline methods, online delivery provides enhanced personalization of interventions tailored to individual characteristics and preferences [44]. This aspect may contribute to further elevating the efficacy of such interventions, as demonstrated by prior research indicating heightened effectiveness when patients were empowered to freely select their habit preferences consistent with their health objectives [39].

Regarding the associations between BCTs and effectiveness of habit formation interventions through meta regression. We identified problem-solving techniques that consistently associated with higher effectiveness of habit formation intervention across analyses and while controlling for significant confounder (viz. follow-up duration in subgroup analysis). Utilizing problem-solving techniques can assist individuals in devising precise action plans to overcome obstacles, thereby enhancing the targeted behavior enactment and making it less vulnerable to relapse [45]. This is particularly crucial, considering that the repetition of behavior within the same context is often fraught with obstacles and repeated failures, due to the complexities and constant changes of daily life [46]. The potential effectiveness of the problem-solving suggests that PA habit may be enhanced when users are provided the service on identifying barriers and developing strategies to overcome them.

Nevertheless, our research has revealed that a subset of BCTs is linked to reduced efficacy of habit formation interventions. Notably, our initial meta-regression analysis found no significant correlation between social reward and a lower effectiveness of habit formation interventions. However, upon further adjusting our model for confounder, a significant correlation emerged. The findings of our study contradict previous research as social reward is believed to have the potential to increase motivation and immediate compliance with a behavior repetitively. However, it is also reported that social reward creates a dependency on external validation and decrease intrinsic motivation [47, 48]. In the context of habit formation interventions, relying on social rewards for motivation and reinforcement may hinder the development of internalized habit strength, as individuals may become more focused on external validation rather than the intrinsic reward of the behavior [49–51]. Based on our research findings, future studies on habit formation interventions may need to delve deeper into the ramifications of extrinsic social rewards on intrinsic rewards, given that intrinsic reward is considered a crucial predictor of habit strength [51].

In conclusion, it is recommended that future habit formation interventions be designed with a consideration of the follow-up duration, the delivery way, and the integration of effective BCTs, to optimize habit formation efficacy.

## Limitations

This study has certain limitations that need to be acknowledged. Firstly, the reliance on self-reported measures of habit strength, specifically the SRHI and SRBAI scales, is a limitation. Self-reported measures can be susceptible to response bias and may not accurately reflect actual behavior change. Additionally, the subjective nature of these measures may lead to variability in interpretation and reporting, which could contribute to the heterogeneity observed in the meta-analysis. Future studies could consider incorporating objective measures of behavior change to complement self-reported measures and enhance the validity of findings.

Secondly, expanding the scope of literature inclusion to encompass other health behaviors, such as dietary habits and meditation practices, is likely to yield different results. Our original plan was to conduct a meta-analysis on a broader range of health behaviors. However, due to the limited number of related studies, a comprehensive synthesis of the effectiveness of habit formation interventions for a wide range of health behaviors cannot be carried out. Future research should consider incorporating a wider range of health-enhancing habits to evaluate the impact of habit formation interventions from a broader perspective.

Thirdly, the coding of BCTs was primarily based on the information provided in the paper, mostly in the methods section, and supporting materials, mostly the experiment protocol, which may not have provided a comprehensive description of all the BCTs used.

Lastly, while we did control for significant confounding variables when analyzing the effects of BCTs, limitations in the number of studies included prevented further control of BCTs, and it was not possible to analyze the optimal dosage or combination of BCTs. Future research should employ a more careful multivariate approach to further examine the relationship between BCTs and the effectiveness of habit formation interventions.

## Conclusions

This systematic review is the first to quantitatively synthesize the effectiveness of habit formation interventions on the automaticity of PA, and further analyze how different study characteristics and BCTs impact intervention effectiveness. The analysis provides evidence for the efficacy of these interventions in promoting PA habits and insights into the association between study design. Future studies could leverage the insights from this study to optimize intervention design and achieve better effectiveness.

### Supplementary Information

**Additional file 1. **

**Additional file 2. **

**Additional file 3. **

## Data Availability

The datasets used and analyzed during the current study are available from the corresponding author on reasonable request.

## Abbreviations

PA: Physical activity
BCT: Behavior change techniques
MeSH: Medical subject headings
SRHI: Self-report habit index
SRBAI: Self-report behavioral automaticity index
SDs: Standard deviations

## References

[1] Hagger MS (2019). Habit and physical activity: Theoretical advances, practical implications, and agenda for future research. Psychol Sport Exerc.

[2] Piercy KL, Troiano RP, Ballard RM, Carlson SA, Fulton JE, Galuska DA (2018). The Physical Activity Guidelines for Americans. JAMA.

[3] Hamilton MT, Healy GN, Dunstan DW, Zderic TW, Owen N (2008). Too little exercise and too much sitting: inactivity physiology and the need for new recommendations on sedentary behavior. Curr Cardiovasc Risk Rep.

[4] Gardner B, de Bruijn G-J, Lally P (2011). A systematic review and meta-analysis of applications of the Self-Report Habit Index to nutrition and physical activity behaviours. Ann Behav Med.

[5] Ajzen I (1991). The theory of planned behavior. Organ Behav Hum Decis Process.

[6] Schwarzer R (2016). Health action process approach (HAPA) as a theoretical framework to understand behavior change. Actualidades en Psicología.

[7] Rhodes RE, de Bruijn GJ (2013). How big is the physical activity intention–behaviour gap? A meta-analysis using the action control framework. Br J Health Psychol.

[8] Armitage CJ, Conner M (2001). Efficacy of the theory of planned behaviour: A meta-analytic review. Br J Soc Psychol.

[9] Rhodes RE, Cox A, Sayar R (2022). What predicts the physical activity intention–behavior gap? A systematic review. Ann Behav Med.

[10] Gawronski B, Creighton LA (2013). Dual process theories.

[11] Orbell S, Verplanken B (2010). The automatic component of habit in health behavior: habit as cue-contingent automaticity. Health Psychol.

[12] Gardner B (2012). Habit as automaticity, not frequency. Eur Health Psychol.

[13] Gardner B, Abraham C, Lally P, de Bruijn G-J (2012). Towards parsimony in habit measurement: Testing the convergent and predictive validity of an automaticity subscale of the Self-Report Habit Index. Int J Behav Nutr Phys Act.

[14] Verplanken B, Orbell S (2003). Reflections on past behavior: a self-report index of habit strength 1. J Appl Soc Psychol.

[15] Feil K, Allion S, Weyland S, Jekauc D (2021). A Systematic Review Examining the Relationship Between Habit and Physical Activity Behavior in Longitudinal Studies. Front Psychol.

[16] Fritz H, Hu Y-L, Gahman K, Almacen C, Ottolini J (2020). Intervention to modify habits: a scoping review. OTJR..

[17] Gardner B, Rebar AL. Habit formation and behavior change. Oxford Research Encyclopedia of Psychology; 2019. 10.1093/acrefore/9780190236557.013.129.

[18] Page MJ, McKenzie JE, Bossuyt PM, Boutron I, Hoffmann TC, Mulrow CD (2021). The PRISMA 2020 statement: an updated guideline for reporting systematic reviews. Int J Surg.

[19] Piao M, Ryu H, Lee H, Kim J (2020). Use of the Healthy Lifestyle Coaching Chatbot App to Promote Stair-Climbing Habits Among Office Workers: Exploratory Randomized Controlled Trial. JMIR Mhealth Uhealth.

[20] Ellingson LD, Lansing JE, DeShaw KJ, Peyer KL, Bai Y, Perez M (2019). Evaluating Motivational Interviewing and Habit Formation to Enhance the Effect of Activity Trackers on Healthy Adults’ Activity Levels: Randomized Intervention. JMIR Mhealth Uhealth.

[21] Higgins JP, Altman DG, Gøtzsche PC, Jüni P, Moher D, Oxman AD (2011). The Cochrane Collaboration’s tool for assessing risk of bias in randomised trials. Bmj..

[22] Cumpston M, Li T, Page MJ, Chandler J, Welch VA, Higgins JP (2019). Updated guidance for trusted systematic reviews: a new edition of the Cochrane Handbook for Systematic Reviews of Interventions. Cochrane Database Syst Rev..

[23] Cohen J, Cohen P, West SG, Aiken LS. Applied multiple regression/correlation analysis for the behavioral sciences. 3rd ed. Taylor and Francis; 2013. 704 p. 10.4324/9780203774441.

[24] Sen S, Yildirim I (2022). A Tutorial on How to Conduct Meta-Analysis with IBM SPSS Statistics. Psych.

[25] Cohen J (2016). A power primer.

[26] Higgins JP, Thompson SG (2002). Quantifying heterogeneity in a meta-analysis. Stat Med.

[27] Michie S, Richardson M, Johnston M, Abraham C, Francis J, Hardeman W (2013). The behavior change technique taxonomy (v1) of 93 hierarchically clustered techniques: building an international consensus for the reporting of behavior change interventions. Ann Behav Med.

[28] Fournier M, Radel R, Bailly L, Pradier C, Fabre R, Fuch A (2018). "As du Coeur" study: a randomized controlled trial on physical activity maintenance in cardiovascular patients. BMC Cardiovasc Disord.

[29] Fleig L, Pomp S, Schwarzer R, Lippke S (2013). Promoting exercise maintenance: how interventions with booster sessions improve long-term rehabilitation outcomes. Rehabil Psychol.

[30] Carels RA, Burmeister JM, Koball AM, Oehlhof MW, Hinman N, LeRoy M (2014). A randomized trial comparing two approaches to weight loss: differences in weight loss maintenance. J Health Psychol.

[31] Storm V, Dörenkämper J, Reinwand DA, Wienert J, De Vries H, Lippke S (2016). Effectiveness of a Web-Based Computer-Tailored Multiple-Lifestyle Intervention for People Interested in Reducing their Cardiovascular Risk: A Randomized Controlled Trial. J Med Internet Res.

[32] Kaushal N, Rhodes RE, Meldrum JT, Spence JC (2018). Mediating Mechanisms in a Physical Activity Intervention: A Test of Habit Formation. J Sport Exerc Psychol.

[33] Rhodes R, Quinlan A, Naylor PJ, Warburton DER, Blanchard CM (2021). Predicting Family and Child Physical Activity across Six-Months of a Family-Based Intervention: an Application of Theory of Planned Behaviour, Planning and Habit. J Sports Sci.

[34] Western MJ, Standage M, Peacock OJ, Nightingale T, Thompson D (2022). Supporting Behavior Change in Sedentary Adults via Real-time Multidimensional Physical Activity Feedback: Mixed Methods Randomized Controlled Trial. JMIR Form Res.

[35] Weyland S, Fritsch J, Feil K, Jekauc D (2022). Investigating the relation between positive affective responses and exercise instigation habits in an affect-based intervention for exercise trainers: A longitudinal field study. Front Psychol.

[36] White I, Smith L, Aggio D, Shankar S, Begum S, Matei R (2017). On your feet to earn your seat: pilot RCT of a theory-based sedentary behaviour reduction intervention for older adults. Pilot Feasibility Stud.

[37] Sawilowsky SS (2009). New effect size rules of thumb. J Mod Appl Stat Methods.

[38] Robinson L, Arden MA, Dawson S, Walters SJ, Wildman MJ, Stevenson M. A machine-learning assisted review of the use of habit formation in medication adherence interventions for long-term conditions. Health Psychol Rev. 2022:1–23. 10.1080/17437199.2022.2034516 35086431

[39] Gardner B, Arden MA, Brown D, Eves FF, Green J, Hamilton K (2023). Developing habit-based health behaviour change interventions: twenty-one questions to guide future research. Psychol Health.

[40] Lally P, Van Jaarsveld CH, Potts HW, Wardle J (2010). How are habits formed: Modelling habit formation in the real world. Eur J Soc Psychol.

[41] Phillips LA, More KR. Evaluating behavior change factors over time for a simple vs. complex health behavior. Front Psychol. 2022;13:962150. 10.3389/fpsyg.2022.962150 PMC9499174 36160596

[42] Sobolev M, Okeke F, Plonsky O, editors. mRAPID Study: Effect of Micro-incentives and Daily Deadlines on Practice Behavior. Persuasive Technology: 18th International Conference, PERSUASIVE 2023, Eindhoven, The Netherlands, April 19–21, 2023, Proceedings; 2023: Springer.

[43] Keller J, Kwasnicka D, Klaiber P, Sichert L, Lally P, Fleig L (2021). Habit formation following routine-based versus time-based cue planning: A randomized controlled trial. Br J Health Psychol.

[44] Alley SJ, Schoeppe S, To QG, Parkinson L, van Uffelen J, Hunt S (2023). Engagement, acceptability, usability and satisfaction with Active for Life, a computer-tailored web-based physical activity intervention using Fitbits in older adults. Int J Behav Nutr Phys Act.

[45] Sullivan AN, Lachman ME (2017). Behavior change with fitness technology in sedentary adults: a review of the evidence for increasing physical activity. Front Public Health.

[46] Segar M (2022). It’s Time to Unhabit and Think Critically About Whether Habit Formation Has Been Over Valued as a Behavior Change Strategy Within Health Promotion.

[47] Lindenberg S (2001). Intrinsic motivation in a new light. Kyklos.

[48] Lepper MR, Greene D, Nisbett RE (1973). Undermining children's intrinsic interest with extrinsic reward: A test of the" overjustification" hypothesis. J Pers Soc Psychol.

[49] Phillips LA, Mullan BA (2023). Ramifications of behavioural complexity for habit conceptualisation, promotion, and measurement. Health Psychol Rev..

[50] Judah G, Gardner B, Kenward MG, DeStavola B, Aunger R (2018). Exploratory study of the impact of perceived reward on habit formation. BMC Psychology.

[51] Kilb M, Labudek S (2022). Effects of behavioral performance, intrinsic reward value, and context stability on the formation of a higher-order nutrition habit: an intensive longitudinal diary study. Int J Behav Nutr Phys Act.

