# Supplementary material for: Effects of habit formation interventions on physical activity habit strength: meta-analysis and meta-regression
Source: Int J Behav Nutr Phys Act. 2023 Sep 12;20:109. doi: 10.1186/s12966-023-01493-3 (PMC10498635; doi:10.1186/s12966-023-01493-3)
Supplement: Supplementary file 2 — Additional file 2. [file 12966_2023_1493_MOESM2_ESM.docx]

PubMed (N=219)

("Habits"[Mesh] OR "Habits"[tiab] OR "Habit"[tiab] OR "habitual"[tiab] OR "SRHI"[tiab]) AND ("Formation"[tiab] OR "Based"[tiab]) AND ("exercise"[Mesh] OR "physical activity"[tiab] OR "Physical Exercises"[tiab] OR "Exercise Training"[tiab]) AND ("randomized controlled trial"[pt] OR "controlled clinical trial"[pt] OR randomized[tiab] OR randomised[tiab] OR placebo[tiab] OR randomly[tiab] OR trial[tiab] OR groups[tiab] )

Embase (N=1452)

('habits'/exp OR 'habits' OR habits:ti,ab OR habit:ti,ab OR habitual:ti,ab OR srhi:ti,ab) AND (formation:ti,ab OR based:ti,ab) AND ('health behavior'/exp OR 'health behavior' OR 'exercise'/exp OR 'exercise' OR ‘physical’ OR activity:ti,ab OR exercises:ti,ab OR 'exercise'/exp OR exercise OR training:ti,ab AND ('randomized controlled trial'/exp OR 'randomized controlled trial' OR 'controlled clinical trial'/exp OR 'controlled clinical trial' OR randomized:ti,ab OR randomised:ti,ab OR placebo:ti,ab OR randomly:ti,ab OR trial:ti,ab OR groups:ti,ab) AND [article]/lim AND [english]/lim AND [2000-2022]/py

**Cochrane CENTRAL (N=1958)**

([mh Habits] OR Habits:ti,ab OR Habit:ti,ab) AND (Formation:ti,ab OR Based:ti,ab) AND (health behavior:ti,ab OR exercise:ti,ab OR 'exercise' OR ‘physical’ OR activity:ti,ab OR exercises:ti,ab OR training:ti,ab)
